# Identifying Bird Remains Using Ancient DNA Barcoding

**DOI:** 10.3390/genes8060169

**Published:** 2017-06-21

**Authors:** Love Dalén, Vendela K. Lagerholm, Johan A. A. Nylander, Nick Barton, Zbigniew M. Bochenski, Teresa Tomek, David Rudling, Per G. P. Ericson, Martin Irestedt, John R. Stewart

**Affiliations:** 1 Department of Bioinformatics and Genetics, Swedish Museum of Natural History, Box 50007, SE-10405 Stockholm, Sweden; love.dalen@nrm.se (L.D.); vendela.lagerholm@st-andrews.ac.uk (V.K.L.); johan.nylander@nrm.se (J.A.A.N.); per.ericson@nrm.se (P.G.P.E.); martin.irestedt@nrm.se (M.I.); 2 Department of Zoology, Stockholm University, SE-10691 Stockholm, Sweden; 3 School of Geography and Sustainable Development, University of St Andrews, North Street, St. Andrews, Fife KY16 9AL, UK; 4 Institute of Archaeology, 36 Beaumont St., Oxford OX1 2PG, UK; nick.barton@arch.ox.ac.uk; 5 Institute of Systematics and Evolution of Animals, Polish Academy of Sciences, Slawkowska 17, 31-016 Krakow, Poland; bochenski@isez.pan.krakow.pl (Z.M.B.); tomek@isez.pan.krakow.pl (T.T.); 6 Sussex School of Archaeology, Mays Farm, Selmeston, Polegate, Sussex BN26 6TS, UK; david.rudling@sussexarchaeology.co.uk; 7 Faculty of Science and Technology, Bournemouth University, Talbot Campus, Fern Barrow, Poole, Dorset BH12 5BB, UK

**Keywords:** Aves, species identification, 16S, palaeogenetics, biodiversity, archaeology, palaeontology

## Abstract

Bird remains that are difficult to identify taxonomically using morphological methods, are common in the palaeontological record. Other types of challenging avian material include artefacts and food items from endangered taxa, as well as remains from aircraft strikes. We here present a DNA-based method that enables taxonomic identification of bird remains, even from material where the DNA is heavily degraded. The method is based on the amplification and sequencing of two short variable parts of the 16S region in the mitochondrial genome. To demonstrate the applicability of this approach, we evaluated the method on a set of Holocene and Late Pleistocene postcranial bird bones from several palaeontological and archaeological sites in Europe with good success.

## 1. Introduction

The ability to rapidly determine the species identity of biological material is important for a multitude of applications, such as in wildlife monitoring, the reconstruction of past floras and faunas in archaeology and palaeontology, as well as the food industry and forensics (e.g., [1,2]). Such species identification is today often done with genetic analysis, utilising comparisons of identified DNA barcodes against reference libraries. This is carried out not only on samples taken directly from the organisms themselves (e.g., tissue fragments, hair samples, and bones), but also on traces of DNA left behind, including faeces [3], urine [4], and footprints [5].

Genetic methods for species identification in animals typically rely on the amplification and sequencing of a ca. 650 base pair long part of the mitochondrial DNA (mtDNA) cytochrome c oxidase 1 gene (COI) [6]. Unfortunately, this standard COI barcode is too long for successful amplification when the DNA in the sample is too degraded [7], which often is the case in forensic and palaeontological remains. The near absence of intermediate conserved regions in the COI gene also makes it problematic to design universal primers that amplify shorter fragments of this barcode gene. Moreover, in cases where samples may contain DNA from several different species (e.g., in faecal material where both predator and prey DNA may be present), more variable regions in the mtDNA genome have often been targeted in order to enable the design of species-specific primers [8]. Thus, several different methods targeting shorter and/or more taxon-specific loci in the mtDNA genome have been developed. However, the taxonomic identification of highly-degraded avian remains is still very challenging, and the currently available markers are either too long or lack sufficient taxonomic resolution or coverage (e.g., [9,10,11]).

The identification of bird remains whose morphology precludes species determination has many applications. Examples include remnants from aircraft bird strikes [12], avian food items, and artefacts recovered by border control. Archaeological and palaeontological fossil sites are a particularly common source of undetermined material. The identification of bird remains from such sites has long been considered challenging [13,14,15,16,17,18], and the species determination of isolated avian bones has traditionally involved the use of morphological characters that can consistently distinguish between taxa (so called non-metric or discrete characters). However, the problem with these methods is that measurements often overlap between closely-related species and that the characters often prove variable [19,20]. Furthermore, the aforementioned approaches rely on the remains being complete enough for morphological comparison, as it is often not possible to identify broken bone fragments, pieces of skin, or other tissues. Some avian taxonomic groups have proven particularly challenging due to the existence of large numbers of closely-related species. The ease with which morphological identification can be made depends on the skeletal element at hand, as some elements are more conservative across related taxa than others. Bones like the coracoid, humerus, carpometacarpus, and tarsometatarsus are considered to be the easiest to identify, although this depends on the taxonomic group to which they belong ([18,21]; authors’ own observations). Other bones that might otherwise be identifiable include crania, but these are not well represented in archaeological and palaeontological records. If a bone is incomplete, it generally requires that an articular end is present for identification to be possible.

One solution to the problem of identifying degraded and/or fragmented bird remains is to use short and diagnostic DNA barcodes. The aim of this study was to develop a method suitable for this purpose, and to demonstrate its applicability through an analysis of bird bones recovered from palaeontological and archaeological sites spanning 125 thousand (k) to 2 k calendar years before present (BP).

## 2. Materials and Methods

### 2.1. Sample Sites

We sampled 25 bones from four palaeontological sites in the United Kingdom and Poland (Table S1). The sites include Joint Mitnor Cave in Devon, England; Merlin’s Cave near Monmouth in Gwent, Wales; Beddingham Roman Villa in Sussex, England; and Oblazowa Cave (Jaskinia w Oblazowej) in the Western Carpathians, Poland.

### 2.2. Design of Avian Barcodes

In order to design and evaluate new barcoding primers, we compiled a taxonomically diverse avian database of the gene encoding for 16S ribosomal RNA in the mitochondrial genome. This gene was chosen as a suitable marker since it is highly conserved throughout most of its sequence; however, in short regions that correspond to loops in the ribosomal structure, the 16S gene is very variable. This property means that it is generally easy to identify primer sites that are conserved across broad taxonomic groups, while at the same time enabling polymerase chain reaction (PCR) amplification of short variable sequences between primer pairs. The 16S also fulfils another important prerequisite for a barcode in that there is extensive reference data available. Our custom-made database consisted of 16S sequences for class Aves downloaded from GenBank (1956 nucleotide sequences on 07/08/2014), combined with locally curated avian 16S sequences (500 nucleotide sequences, generated at the Swedish Museum of Natural History and available from Martin Irestedt or Per Ericson on request), and included representatives from almost all avian families that occur in Europe (families in Galloanserae to families in Neoaves). To develop general bird primers with good resolution that could be used to amplify highly degraded DNA, we identified the most conserved regions in the reference dataset which flanked variable loops. Primer pairs were then designed around two short variable stretches of the 16S gene (the regions are herein referred to as Aves-16S-1A and Aves-16S-2A; Table 1). The integrity of the new primers was tested using the program Amplify (available from http://engels.genetics.wisc.edu/amplify/) to ensure that they did not have alternative binding sites or formed primer dimers. Since a specificity test showed that one of the primer pairs (for fragment Aves-16S-1A) also amplifies human DNA (a common contaminant in ancient DNA analyses), we also designed a human blocking primer containing a C3-spacer for this fragment (Table 1).

### 2.3. Laboratory Procedures

Approximately 20 mg of bone powder was obtained using a Dremel tool (Dremel, Breda, The Netherlands) operated at low speed to minimize temperature-induced loss of DNA. This is a comparatively small amount of bone to sacrifice through destructive sampling and most of the diagnostic anatomical features can still be preserved (see Figure S1). DNA was extracted using a silica-based method, following the protocol described in Ersmark et al. [22]. PCRs were carried out in 25 μL volumes containing 1× PCR buffer, 2.5 mM MgCl_2_, 0.1 mg/mL BSA, 0.2 mM of each dNTP, 2 U Hotstar Taq (Qiagen, Hilden, Germany), 0.2 μM of each avian primer, 2 μM of human blocking primer (where applicable), and 2 μL of extracted DNA. The PCRs were run using a 10 min denaturation step at 95 °C, followed by 55 cycles comprising 30 s at 94 °C, 30 s at 52 °C, 30 s at 72 °C, and a final 7 min elongation step at 72 °C. The resulting PCR products were checked on a 2% agarose gel, and the successful amplifications were cleaned using Exo-FAP (Fermentas, Vilnius, Lithuania). Following clean-up, both forward and reverse strands were sequenced using an ABI 3130xl sequencer (Applied Biosystems, Foster City, CA, USA).

To minimise contamination, DNA extraction and PCR-setup were done in a dedicated ancient DNA facility, employing standard precautions such as sterilization of equipment and reagents using 0.5% sodium hypochlorite solutions or UV light. Negative controls were included at both the extraction and amplification stages in order to monitor for possible contamination. Moreover, the PCRs were replicated to identify erroneous bases caused by DNA degradation.

### 2.4. Data Analyses

Our 16S database was used as reference library in the sequence identifications, employing the stand-alone blastn algorithm as implemented in the BLAST+ software suite [23]. Default values were used for blastn (E = 10, word size = 11, gapopen = 5, gapextend = 2), and for a positive identification, we required a high similarity (>97%) with minimum mismatches over the matching region.

## 3. Results and Discussion

Out of the 25 samples analysed in the study, 18 were successfully sequenced for both fragments that were targeted. The oldest sample—from Joint Mitnor Cave dated to the last interglacial (the Eemian, 130–116 k years BP)—did not produce any successful result. This is not too surprising, given the site’s extreme age of ca. 125 k years. Overall, the genetic similarity between sequenced samples and database sequences was very high (97.5–100, median 100%, with a maximum of one mismatch over the aligned regions), thus allowing species assignments with high confidence (Table S2). The identifications corresponded well with the a priori morphological classifications done on each of the bone samples (Table 2). However, in a few instances, the species determination done in the genetic analyses differed from the morphological ones, and some of these are interesting to discuss further. The identities from the sites of progressively younger age are as follows. 

Oblazowa Cave is Late Glacial, with the layers that provided material for this study being dated to between 13 k BP and 29 k BP [23]. We genetically identified fieldfare *Turdus pilaris*, blackbird *Turdus merula*, skylark *Alauda arvensis*, and shorelark *Eremophila alpestris*. The main discrepancy between the morphological identifications and that from the ancient DNA analyses is the shorelark formerly identified as a wood lark. Wood lark may not have been out of the realms of possibility, because although its range today is that of a temperate bird [24], species with similar distributions are known from the last glacial in Britain in what have been described as non-analogue communities [25]. However, shorelark is a bird breeding in the tundra of the Palaearctic today, and this fits well with a Late Glacial age accompanying birds like the here-identified fieldfare and commonly associated taxa such as rock ptarmigan and the Norwegian lemming. Blackbird and skylark are currently found across most of Scandinavia up to the northernmost areas, albeit as breeding migratory populations [24]. Therefore, these species are not unexpected for a Late Glacial site.

The Merlin’s Cave assemblage—which probably dates from the Late Glacial to Early Holocene—includes identification of rock ptarmigan *Lagopus muta* and fieldfare *Turdus pilaris*, which are both common bird species in north-western European Late Glacial sites [16,26,27]. Another expected bird is the jackdaw *Corvus monedula*, which is often associated with nesting on rock faces [28] and is relatively cold-tolerant [24]. On the other hand, the identified corn bunting *Emberiza calandra* has a modern distribution characteristic of a temperate species, whose northern limits today are Scotland, Denmark, the southern tip of Sweden, and Latvia [29]. The corn bunting may therefore represent a non-analogue association, although it is a species of open grassland which is consistent with the Late Glacial. However, the age of the material from Merlin’s Cave is imprecise, and if the specimen is Early Holocene, it would explain the presence of this temperate species. The surprising results were the two bones genetically identified as a wheatear in the *Oenanthe* complex, with a best match to the East African species *Oenanthe lugubris* [30,31]. The phylogeny of this group is not completely resolved, though, and more data such as an in-depth analysis of genetic variation in wheatears using additional DNA markers would be needed to confirm the finding. However, if this holds up, it would mean that the original taxonomic classifications of the two bones (tentatively to the family Fringillidae or Emberizidae without using the exhaustive methods detailed in Stewart and Jacobi [23]; Table 2) were incorrect up to the genus and family levels. This error could be partly explained by the fact that the proximal shape of wheatear humeri are more superficially similar to those of finches and buntings than would be expected based on their respective phylogenetic positions, as shown by a morphological review of the sampled specimens. In addition, the identity revealed using ancient DNA is unlikely to have been accomplished using morphology, because researchers would not generally have considered the taxon recognised here as a possible candidate. Apart from the overall difficulty in identifying bird remains [17,19], there is an unspoken belief that possible species would be found in the same zoogeographic province as the find locality [32]. 

Assuming it to be correct, the identification of a species in Late Glacial Europe that today is a non-migratory species native to Africa might seem surprising. One could speculate that it relates to the hypothesis that sedentary populations of birds sometimes evolve from migratory ones [19], and that the bones from Merlin’s cave belonged to a migratory population that used to breed in Britain. Whether it belonged to an extant *Oenanthe* species that has since developed a sedentary southern lifestyle, or a separate evolutionary lineage of wheatears that is now extinct, is not possible to resolve with the limited genetic data currently available. There are, however, many examples where avian species or populations appear to have evolved from being migratory to sedentary [33]. For instance, the sedentary southern European spotless starling (*Sturnus unicolor*) probably evolved from the largely migratory common starling (*Sturnus vulgaris*) [19], and similar evolutionary processes have also been revealed for the monarch butterfly (*Danaus plexippus*) [34].

Beddingham Roman Villa, with contexts dated by pottery seriation to the 1st–4th centuries AD, include remains molecularly identified as coming from domestic fowl (*Gallus gallus*), which is expected in a Roman villa site [35], as they were part of the Roman diet and had been introduced to Britain during the Iron Age [36]. The other identifications include birds of farmland (corn bunting *Emberiza calandra*), forest edge and hedgerow (blackbird *Turdus merula* and song thrush *Turdus philomelos*), wetlands (Eurasian wigeon *Anas penelope*) [24] and taxa that may have been domesticates (greylag goose *Anser anser* and rock dove *Columbia livia*). The morphological misidentification of the rock dove as a tentative wood pigeon (which is larger [37]) can be accounted for by the fact that no actual comparison by measurement had been achieved, and instead the size was judged by eye. The erroneous classification of the greylag goose as a domestic fowl is explained by the fragmentary nature of the bone—a tibiotarsus shaft—and the close morphological similarity and size for these otherwise disparate birds. The wigeon is interesting, because in southern England today this species is a migratory winter visitor [24], and without molecular evidence, their remains are likely to be mistaken for resident mallard (*Anas platyrhynchos*) or other similarly sized ducks in the genus *Anas* or even other duck genera [38].

## 4. Conclusions

For the purpose of this study, we intentionally analysed more or less complete bones that were often diagnostic for inter-specific morphological comparisons. As a result, the taxonomic assignments based on ancient DNA barcoding were in general agreement with the original morphological identifications. However, the vast majority of avian bones in prehistoric sites are heavily fragmented, or represent skeletal elements such as phalanges and vertebrae, which are unsuitable for morphological species determination. It should also be noted that preservation and certainly the recovery on site of diagnostic bones might be biased towards bird species with larger body size, and the taxonomic identifications may also be influenced by researchers’ expectations based on current species distributions (as exemplified by the surprising find of an African wheatear in Merlin’s cave). Thus, the molecular method presented here opens up for much more comprehensive and likely less biased analyses of avian species assemblages within paleontological and archaeological sites, and across horizons within sites. The barcoding method should also provide a useful initial approach for future ancient DNA studies on specific bird species, since it offers a way to easily scan unclassified avian collections for samples that can be genetically verified as both belonging to the species under investigation and containing preserved DNA. Thus, palaeontologists interested in cross-disciplinary research projects would not need to provide geneticists with valuable diagnostic remains, but can offer less important fragments for destructive sampling.

The protocol presented here should also be relatively straightforward to scale up through the use of high-throughput sequencing on pooled amplicons. A combination of barcoded PCR primers [39] and indexed sequencing adapters could permit thousands of samples to be analysed on one single sequencing run. This would provide a means to very rapidly estimate prehistoric avian species compositions, which would enable detailed estimates of temporal changes in community composition (e.g., across past changes in climate).

Finally, this research has once again demonstrated the power of ancient DNA methods in producing unexpected identifications of Quaternary vertebrates. The most notable being the previous discovery of the Denisovans, the distinct human population found in Siberia [40]. We should probably expect further surprises among the relatively understudied birds of the Pleistocene.

## Figures and Tables

**Table 1:** Developed barcoding primer pairs, including the human blocking primer.

Primer	Sequence (5’-3’)	Amplicon Size
Aves-16S-1AF	CATAAGACGAGAAGACCCTGTGGA	c. 125 bp
Aves-16S-1AR	TCCAAGGTCGCCCCAACCGAA	
Aves-16S-2AF	CCTTGGAGAAAAACAAANCCTCCAAA	c. 120 bp
Aves-16S-2AR	TCCCTGGGGTAGCTTGGTCCAT	
Aves-16S-1A-Block	AGACCCTATGGAGCTTTAATTTATTAATGCAAAC	

bp: base pairs.

**Table 2:** Taxonomic assignment of samples based on ancient DNA, compared to previous morphological identifications. See Table S1 for more detailed sample information.

Sample ID	Genetic Assignment	Morphological Assignment
J2	*Oenanthe lugubris*	Fringillidae/Emberizidae (Size of *Emberiza calandra*, *Pinicola enucleator*, *Coccothraustes coccothraustes*)
J3	*Oenanthe lugubris*	Fringillidae/Emberizidae (Size of *E. calandra*, *P. enucleator*, *C. coccothraustes*)
J4	*Turdus pilaris*	*Turdus* sp.-*T.* cf. *pilaris/viscivorus*
J5	*Turdus merula*	*Turdus* sp.-*T*. cf. *merula*
J6	*Emberiza calandra*	Fringillidae/Emberizidae (Size of *E. calandra*, *P. enucleator*, *C. coccothraustes*)
J7	*Turdus philomelos*	*Turdus* sp.-*T*. cf. *philomelos*
J8	*Emberiza calandra*	Passeriformes (Size between Blackbird and House sparrow)
J9	*Anser anser*	cf. *Gallus gallus*
J10	*Gallus gallus*	cf. *Gallus gallus*
J11	*Columba livia*	Columbiformes (Looks approx. *Columba palumbus* size)
J12	*Gallus gallus*	cf. *Gallus gallus*
J13	*Anas penelope*	Anatinae (ca. Mallard size)
J14	*Corvus monedula*	Corvidae (*Corvus monedula* size)
J17	*Lagopus muta*	Aves, size of *Lagopus*?
J19	*Turdus pilaris*	*Turdus* sp.
J20	*Turdus pilaris*	*Turdus viscivorus*
J21	*Turdus merula*	*Turdus* sp. (size *T. merula*)
J22 *	*Alauda arvensis*	*Alauda arvensis*
J24 *	*Eremophila alpestris*	*Lullula arborea*
J25	*Eremophila alpestris*	*Lullula arborea*

cf: compare; suggestion of possible identity. * Species identification based on the second DNA fragment only.

## Supplementary Materials

The following are available online at www.mdpi.com/2073-4425/8/6/169/s1. Table S1: Identity of the sampled avian bones, Table S2: Output from BLAST+ showing the best taxon match for the successful ancient DNA sequences against the custom database. Figure S1: Photo of the drilled humerus bone from specimen J1, illustrating the amount of material that is needed for ancient DNA analyses.
