# Supplementary material for: Identifying Bird Remains Using Ancient DNA Barcoding
Source: Genes (Basel). 2017 Jun 21;8(6):169. doi: 10.3390/genes8060169 (PMC5485533; doi:10.3390/genes8060169)
Supplement: Supplementary file 1 [file genes-08-00169-s001.docx]

Supplementary information for:

**Identifying bird remains using ancient DNA barcoding**

**Love Dalén, Vendela K. Lagerholm, Johan A. A. Nylander, Nick Barton,** [**Zbigniew M. Bochenski**](http://www.sciencedirect.com/science/article/pii/S0305440308001799)**, Teresa Tomek, David Rudling, Per G. P. Ericson, Martin Irestedt and John R. Stewart**

**Table S1.**  Identity of the sampled avian bones. Approximate ages are denoted as Eemian, Late Glacial (LG), Early Holocene (EH) and Roman.

| **Lab ID** | **Specimen label** | **Site** | **Material** | **Age** | **Identification** |
| --- | --- | --- | --- | --- | --- |
| J1 | MER (9) TR A, Spit 5 | Merlin's Cave, UK | Right humerus | LG/EH ^1^ | J.S |
| J2 | MER (9) TR A, Spit 5 | Merlin's Cave, UK | Proximal left humerus | LG/EH ^1^ | J.S |
| J3 | MER (9) TR A, Spit 5 | Merlin's Cave, UK | Right humerus | LG/EH ^1^ | J.S |
| J4 | MER (8) TR A, Spit 4 | Merlin's Cave, UK | Carpometacarpus | LG/EH ^1^ | J.S |
| J5 | 1987.2 /I/ (972) | Beddingham Villa, UK | Right humerus | Roman ^2^ | J.S |
| J6 | 1987.2 /I/ (14) | Beddingham Villa, UK | Right humerus | Roman ^2^ | J.S |
| J7 | 1987.2 /I/ (101) | Beddingham Villa, UK | Tarsometatarsus | Roman ^2^ | J.S |
| J8 | MER (2) W. front initial clean | Merlin's Cave, UK | Distal left humerus | LG/EH ^1^ | J.S |
| J9 | 1987.2 /I/ (367) | Beddingham Villa, UK | Left tibiotarsus shaft | Roman ^2^ | J.S |
| J10 | 1987.2 /I/ (367) | Beddingham Villa, UK | Proximal right carpometacarpus | Roman ^2^ | J.S |
| J11 | 1987.2 /I/ (367) | Beddingham Villa, UK | Distal left carpometacarpus | Roman ^2^ | J.S |
| J12 | 1987.2 /I/ (79) | Beddingham Villa, UK | Left humerus (juvenile) | Roman ^2^ | J.S |
| J13 | 1987.2 /I/ (79) | Beddingham Villa, UK | Left tarsometatarsus shaft | Roman ^2^ | J.S |
| J14 | MER (8) TR A, Spit 4 | Merlin's Cave, UK | Right coracoid fragment | LG/EH ^1^ | J.S |
| J15 | MER (8) TR A, Spit 4 | Merlin's Cave, UK | Right ulna | LG/EH ^1^ | J.S |
| J16 | MER (8) TR A Spit 7 | Merlin's Cave, UK | Right coracoid fragment | LG/EH ^1^ | J.S |
| J17 | MER (8) TR A Spit 7 | Merlin's Cave, UK | Synsacrum fragment | LG/EH ^1^ | J.S |
| J18 | JM 96, TC2, Area A, Spit 5 | Joint Mitnor, UK | Coracoid fragment | Eemian ^3^ | J.S |
| J19 | AF OBZ/649, Layer II | Oblazowa Cave, Poland | Right tarsometatarsus | LG ^4^ | T.T |
| J20 | AF OBZ/610, Layer IV | Oblazowa Cave, Poland | Left carpometacarpus | LG ^4^ | T.T |
| J21 | AF OBZ/602, Layer IV-VI | Oblazowa Cave, Poland | Distal left carpometacarpus | LG ^4^ | Z.B |
| J22 | AF OBZ/450, Layer IV | Oblazowa Cave, Poland | Left carpometacarpus | LG ^4^ | T.T |
| J23 | AF OBZ/459, Layer II | Oblazowa Cave, Poland | Distal left humerus | LG ^4^ | T.T |
| J24 | AF OBZ/483, Layer II | Oblazowa Cave, Poland | Proximal right ulna | LG ^4^ | T.T |
| J25 | AF OBZ/482, Layer II | Oblazowa Cave, Poland | Right ulna | LG ^4^ | T.T |

^1^ Mean of published dates from the same site is 11 k BP [[1](#_ENREF_1)]. ^2^ Rudling [[2](#_ENREF_2)]. ^3^ The Eemian in Britain is dated to 125 k BP [[3](#_ENREF_3)]. ^4^ Published dates from the sampled layers are 13 k BP (layer II) and 18 k BP and 29 k BP (layer V) [[4](#_ENREF_4)].

**Table S2**. Output from BLAST+ showing the best taxon match for the successful ancient DNA sequences against the custom database.

| **Query id** | **Binomen** | **Subject id** | **% Identity** | **Alignment length** | **Mis-matches** | **Gap opens** | **q. start** | **q. end** | **s. start** | **s. end** | **evalue** | **Bit score** |
| --- | --- | --- | --- | --- | --- | --- | --- | --- | --- | --- | --- | --- |
| J2 | Oenanthe lugubris * | gi\|300432064\|gb\|HM046851.1\| Oenanthe schalowi isolate 447 16S ribosomal RNA gene, partial sequence; mitochondrial | 100 | 74 | 0 | 0 | 119 | 192 | 323 | 396 | 1E-33 | 137 |
| J3 | Oenanthe lugubris * | gi\|300432064\|gb\|HM046851.1\| Oenanthe schalowi isolate 447 16S ribosomal RNA gene, partial sequence; mitochondrial | 100 | 74 | 0 | 0 | 119 | 192 | 323 | 396 | 1E-33 | 137 |
| J4 | Turdus pilaris | Turdus_pilaris_NRM20066901_16S.seq "Contig 23" (1,549) | 98.7 | 78 | 1 | 0 | 1 | 78 | 212 | 289 | 9E-35 | 141 |
| J5 | Turdus merula | Turdus_merula_NRM20056091_16S.seq "Contig 42" (1,549) | 100 | 58 | 0 | 0 | 135 | 192 | 347 | 404 | 9E-25 | 108 |
| J6 | Emberiza calandra | Emberiza_calandra_NRM20046026_16S.seq "Contig 5" (1,550) | 100 | 78 | 0 | 0 | 1 | 78 | 211 | 288 | 7E-36 | 145 |
| J7 | Turdus philomelos | Turdus_philomelos_NRM976168_16S.seq "Contig 13" (1,550) | 100 | 74 | 0 | 0 | 119 | 192 | 331 | 404 | 1E-33 | 137 |
| J8 | Emberiza calandra | Emberiza_calandra_NRM20046026_16S.seq "Contig 5" (1,550) | 100 | 78 | 0 | 0 | 1 | 78 | 211 | 288 | 7E-36 | 145 |
| J9 | Anser anser | gi\|544582183\|gb\|KC984218.1\| Anser anser 16S ribosomal RNA gene, partial sequence; mitochondrial | 97.5 | 80 | 1 | 1 | 1 | 80 | 7 | 85 | 1E-33 | 137 |
| J10 | Gallus gallus | gi\|29824878\|gb\|AY236430.1\| Gallus gallus 16S ribosomal RNA gene, partial sequence; mitochondrial gene for mitochondrial product | 100 | 78 | 0 | 0 | 1 | 78 | 22 | 99 | 7E-36 | 145 |
| J11 | Columba livia | Columba_livia_NRM20076011_16S.seq "Contig 1" (1,546) | 100 | 75 | 0 | 0 | 1 | 75 | 210 | 284 | 3E-34 | 139 |
| J12 | Gallus gallus | gi\|29824878\|gb\|AY236430.1\| Gallus gallus 16S ribosomal RNA gene, partial sequence; mitochondrial gene for mitochondrial product | 98.7 | 79 | 0 | 1 | 1 | 79 | 22 | 99 | 3E-34 | 139 |
| J13 | Anas penelope | Anas_penelope_NRM20036435_16S.seq "Contig 3" (1,557) | 100 | 79 | 0 | 0 | 1 | 79 | 211 | 289 | 2E-36 | 147 |
| J14 | Corvus monedula | Corvus_monedula_NRM986450_16S.seq "Contig 7" (1,548) | 100 | 77 | 0 | 0 | 1 | 77 | 212 | 288 | 2E-35 | 143 |
| J17 | Lagopus muta | Lagopus_muta_NRM986101_16S.seq "Contig 52" (1,554) | 100 | 76 | 0 | 0 | 115 | 190 | 328 | 403 | 8E-35 | 141 |
| J19 | Turdus pilaris | Turdus_pilaris_NRM20066901_16S.seq "Contig 23" (1,549) | 100 | 74 | 0 | 0 | 118 | 191 | 331 | 404 | 1E-33 | 137 |
| J20 | Turdus pilaris | Turdus_pilaris_NRM20066901_16S.seq "Contig 23" (1,549) | 100 | 78 | 0 | 0 | 1 | 78 | 212 | 289 | 7E-36 | 145 |
| J21 | Turdus merula | Turdus_merula_NRM20056091_16S.seq "Contig 42" (1,549) | 100 | 78 | 0 | 0 | 1 | 78 | 212 | 289 | 7E-36 | 145 |
| J22_(frag2) | Alauda arvensis | Alauda_arvensis_NRM996263_16S.seq "Contig 4" (1,552) | 98.7 | 74 | 1 | 0 | 1 | 74 | 331 | 404 | 5E-33 | 134 |
| J24_(frag2) | Eremophila alpestris | gi\|220900200\|gb\|FJ465221.1\| Eremophila alpestris albigula voucher MFUM 20042 16S ribosomal RNA gene, partial sequence; mitochondrial | 100 | 74 | 0 | 0 | 1 | 74 | 296 | 369 | 4E-34 | 137 |
| J25 | Eremophila alpestris | gi\|220900200\|gb\|FJ465221.1\| Eremophila alpestris albigula voucher MFUM 20042 16S ribosomal RNA gene, partial sequence; mitochondrial | 100 | 80 | 0 | 0 | 1 | 80 | 175 | 254 | 5E-37 | 148 |

* A corrigendum to the associated publication has stated that the deposited *Oenanthe schalowi* sequences were derived from incorrectly labelled specimens, and actually belong to the species *O. lugubris* [[5](#_ENREF_5),[6](#_ENREF_6)].

**
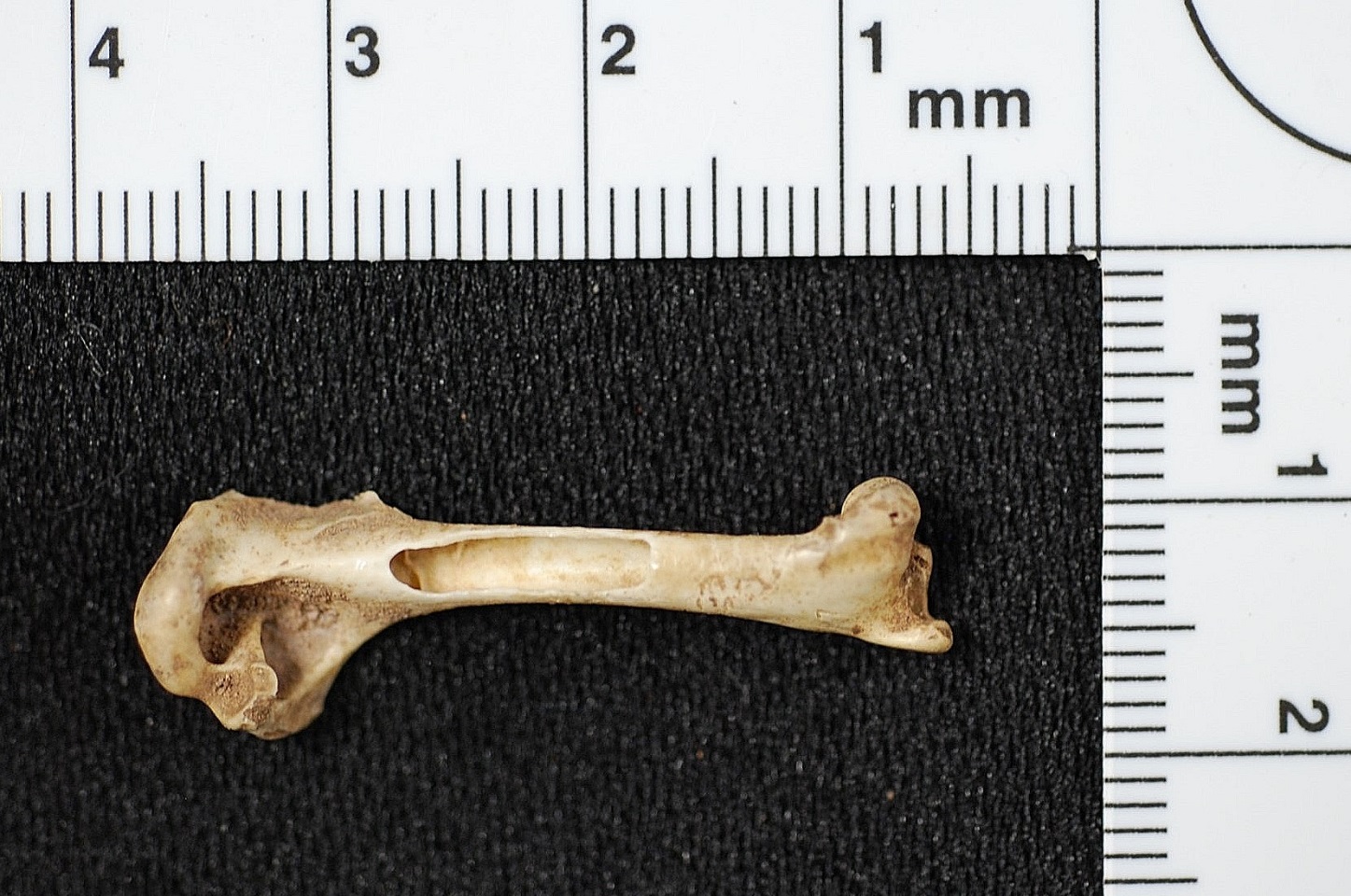
Figure S1**. Photo of the drilled humerus bone from specimen J1, illustrating the amount of material that is needed for ancient DNA analyses.

**References**

1. Ramsey, C.B.; Higham, T.F.G.; Owen, D.C.; Pike, A.W.G.; Hedges, R.E.M. Radiocarbon dates from the Oxford AMS System: archaeometry datelist 31. *Archaeometry* **2002**, *44*, 1-149.

2. Rudling, D. A Tale of Two Villas: Beddingham and Barcombe. *Bulletin of the Association for Roman Archaeology* **2003**, 10-15.

3. Currant, A.; Jacobi, R. A formal mammalian biostratigraphy for the Late Pleistocene of Britain. *Quat. Sci. Rev.* **2001**, *20*, 1707-1716.

4. Bochenski, Z.; Bochenski, Z.M.; Tomek, T. *A history of Polish birds*. Institute of Systematics and Evolution of Animals, Polish Academy of Sciences: Krakow, 2012.

5. Förschler, M.I.; Khoury, F.; Bairlein, F.; Aliabadian, M. Phylogeny of the mourning wheatear *Oenanthe lugens* complex. *Molecular Phylogenetics and Evolution* **2010**, *56*, 758-767.

6. Förschler, M.I.; Khoury, F.; Bairlein, F.; Aliabadian, M. Corrigendum to “Phylogeny of the mourning wheatear *Oenanthe lugens* complex” [Mol. Phylogenet. Evol. 56 (2010) 758–767]. *Molecular Phylogenetics and Evolution* **2010**, *57*, 483-484.
